# Medical History from the Earliest Times

**Published:** 1893-06-24

**Authors:** E. T. Withington


### Medical History from the Earliest Times.

By E. T. Withington, M.A., M.B. Oxon.

### LIX.—THE ANIMISTS.

The eighteenth or philosophic century, as it has been called, is marked in medical history by the rise and fall of a number of philosophic theories and systems, and by a revival of that Platonic mysticism which we have more than once noticed as the evil genius of the healing art. We find abstract expressions treated as though they were real existences, and attempts made to solve the problems of life and disease by such terms as "vital principle," "excitability," and "polarity," explanations no more scientific and much less poetical than that of thunder as the voice of Jove, or diseases as the arrows of Apollo.

The chief exponents of the so-called vitalistic systems of medicine may be divided into two classes, a metaphysical and a scientific. The former held that the body is composed of passive or "dead" matter, and is inhabited by a mysterious immaterial being called "life," which acts upon the body as it were from without, and separates from it at death. And they may be subdivided into two sections: (1) the animists, who identified this life with the soul, and (2) the pure vitalists, who maintained the existence of a second mysterious entity, the "vital principle." In contrast to both of these, the organicists (as they may be called) taught that vital activity is the result of the intimate structure of organic matter—that life, in short, is the effect not the cause of organisation; but they differed from the materialists of the preceding age in holding that vital forces are entirely separate from, and in a sense superior to, those of physics and chemistry. Space only permits us to consider, in the present chapter, the typical representative of the first sub-division of the former class, George Ernest Stahl, professor at Halle, a name equally famous in the history of medicine and of chemistry.

Stahl (1660-1734) whose deep piety and considerable genius were clouded by an unfortunate temper, and an obscure style, tells us that when he began to teach, there were no doctors, "for medicine is the science of life, and we have now only mechanics and chemists." "I deny (he says), that chemistry has anything to do with medicine." "Where the physicist ends, the physician begins," and he even declares that anatomy, except in its broader outlines, is useless to the healing art. The animal body consists of highly putrescent substances, but does not putrefy while alive. Life, therefore, is something which resists putrefaction; and it does this, according to Stahl, by keeping the blood, the most putrescent part, in continual motion, and by expelling whatever is beginning to corrupt by the secretions and excretions. Motion in itself is immaterial, and presupposes an immaterial agent, and Stahl declares that the source of all vital movement is nothing else than the soul, which builds up the machine of the body, and maintains it for a time against external influences. The hypothesis of a special force called nature, pneuma, archeus, &c., is an old wives' fable (antiqua nænia), for if unintelligent how is it superior to mere mechanism, if intelligent, why distinguish it from the rational soul? The objection that spirit cannot act upon matter without some intermediary refutes itself, for this intermediary must be spiritual or material: in the first case it cannot act upon the body, in the second it cannot be acted on by the soul. He then supports his own theory by a great array of facts showing the close connection of mind and body, some of which will readily occur to the reader, and which might nowadays be considerably extended, for the "anima" of Stahl closely resembles the sub-conscious personality of modern hypnotism.

One of the difficulties of animism is the explanation of natural death. The substance of the body is continually renewed; if the force behind it is an immortal soul, why should it not go on for ever? Stahl faces the problem boldly; he declares that the immediate cause of death is not disease, but the direct action of the soul, and he quotes with approval the curious expression of Seneca, "You die not because you are ill, but because you are alive." The soul leaves the bodily machine either because it has become unworkable through some serious lesion, or because it does not choose to work it any longer; but he attempts to justify this suicide by asserting that the tendency to putrefaction increases with age, and may finally become irresistible.

As a rule, the soul tries to preserve the body as long as possible, and most so-called diseases are merely manifestations of its efforts in this direction. Such is the case, for instance, with the whole class of fevers. The natural tendency of the blood to putrefy has somehow become increased; the soul perceiving this, at once counteracts it by more rapid circulation and excretion; and Stahl observes that man is more liable to febrile diseases than are animals, because his more intelligent soul sees and guards against approaching dangers which the stupid "animæ" of brutes do not perceive; therefore they have fewer illnesses but shorter lives. The most fertile cause of disease is plethora, which the soul relieves by natural losses of blood, by the nose in childhood, by the lungs in youth, and by the hæmorrhoidal or uterine veins in adults. The suppression of this natural loss causes headache, impetigo, and other head affections in children, pulmonary diseases in the young, and hysteria, hypochondria, renal and vesical affections in older persons. The chief source of chronic disease is the portal system, where the circulation is most sluggish, and therefore the natural tendency to putrefaction least counteracted, and Stahl wrote a special treatise, "De venæ portæ porta malorum," on the multifarious evils of "portal congestion," long afterwards a favourite phrase with physicians at a loss for a diagnosis. But the soul is liable to error in its sub-conscious as well as in its conscious activities, and some diseases are due to a false idea or irregular motion (motus ataxiæ) on its part. It may, for instance, send an excess of blood to the lungs, and so produce congestion, stagnation, and their infallible results, putrefaction, and destruction of tissue, in short, pulmonary phthisis; a doctrine which reminds us of the "custos errans" of Van Helmont.

Stahl's therapeutics, as might be expected, were of the simplest character. The soul, in spite of its mistakes, knows much more about the body it has built up than does the most skilful physician. The latter's chief duty, therefore, is to watch and assist its efforts. Venesection and purgation are the great remedies for the numerous ills of plethora; copious water drinking dilutes the blood, and favours its free circulation, and some aromatic substances may assist the soul in its great object of warding off corruption. But in his old age Stahl gave up even this simple medication. Fevers, he declared, are not to be treated at all, unless we can discover and remove their cause. He therefore rejected quinine. Opium, also, is most harmful, for it tends to deprive the soul of its control over the body; and he finally contented himself with dosing all his patients with salt and water, which could not do much harm, and might help to counteract putrefaction.

Such, in briefest outline, were the doctrines of "animism," a system originating partly in a reaction against the materialism of the preceding age, partly in the attempt of a pious and philosophic physician to reconcile medicine and theology. But Stahl, like most conciliators, satisfied neither party. The theologians objected to the soul being degraded into a sort of sanitary inspector who goes about the body, cleaning out drains, removing refuse, &c., and sometimes performs even those humble duties imperfectly. And they objected still more to his theory of "natural" death. In old age there is certainly a decay of vital force, and we sometimes seem able to trace its gradual and complete extinction. To admit that this vital force is the soul would be to place a dangerous weapon in the hands of those who deny its immortality, and the theologians therefore treated Stahl as a Philistine, and sent out their champion philosopher, Leibnitz, to smite him. To the more materialistic physicians Stahl's doctrine of a soul, immaterial and indivisible, without extension in space, yet present in and acting upon every part of the body at the same time, seemed the very *ne plus ultra* of absurdity; while those who cared less for theory than for practice found little to attract them in a system the therapeutic part of which bordered upon absolute Nihilism. Still, the doctrine of Stahl is of much importance, if only as marking the close of a materialistic and the beginning of a metaphysical epoch in medicine, and the animists and semi-animists included several able physicians, some of whom may be noticed in the sequel.

